# Augmenting Clinical Outcome Measures of Gait and Balance with a Single Inertial Sensor in Age-Ranged Healthy Adults

**DOI:** 10.3390/s19204537

**Published:** 2019-10-18

**Authors:** Megan K. O’Brien, Marco D. Hidalgo-Araya, Chaithanya K. Mummidisetty, Heike Vallery, Roozbeh Ghaffari, John A. Rogers, Richard Lieber, Arun Jayaraman

**Affiliations:** 1 Max Nader Lab for Rehabilitation Technologies and Outcomes Research, Shirley Ryan AbilityLab, Chicago, IL 60611, USA; mobrien02@sralab.org (M.K.O.); marhiar@gmail.com (M.D.H.-A.); cmummidise@sralab.org (C.K.M.); 2 Department of Physical Medicine and Rehabilitation, Northwestern University, Chicago, IL 60611, USA; 3 Department of BioMechanical Engineering, Delft University of Technology, 2628CD Delft, The Netherlands; H.Vallery@tudelft.nl; 4 Shirley Ryan AbilityLab, Chicago, IL 60611, USA; rlieber@sralab.org; 5 Center for Bio-Integrated Electronics, Departments of Materials Science and Engineering, Biomedical Engineering, Electrical Engineering and Computer Science, Northwestern University, Evanston, IL 60208, USA; rooz@northwestern.edu (R.G.); jrogers@northwestern.edu (J.A.R.)

**Keywords:** wearable sensors, rehabilitation, gait events, gait impairment, postural sway, fall risk, Ten-Meter Walk Test, Berg Balance Scale, Timed Up and Go

## Abstract

Gait and balance impairments are linked with reduced mobility and increased risk of falling. Wearable sensing technologies, such as inertial measurement units (IMUs), may augment clinical assessments by providing continuous, high-resolution data. This study tested and validated the utility of a single IMU to quantify gait and balance features during routine clinical outcome tests, and evaluated changes in sensor-derived measurements with age, sex, height, and weight. Age-ranged, healthy individuals (N = 49, 20–70 years) wore a lower back IMU during the 10 m walk test (10MWT), Timed Up and Go (TUG), and Berg Balance Scale (BBS). Spatiotemporal gait parameters computed from the sensor data were validated against gold standard measures, demonstrating excellent agreement for stance time, step time, gait velocity, and step count (intraclass correlation (ICC) > 0.90). There was good agreement for swing time (ICC = 0.78) and moderate agreement for step length (ICC = 0.68). A total of 184 features were calculated from the acceleration and angular velocity signals across these tests, 36 of which had significant correlations with age. This approach was also demonstrated for an individual with stroke, providing higher resolution information about balance, gait, and mobility than the clinical test scores alone. Leveraging mobility data from wireless, wearable sensors can help clinicians and patients more objectively pinpoint impairments, track progression, and set personalized goals during and after rehabilitation.

## 1. Introduction

Gait and balance play a vital role in functional mobility. From a clinical perspective, these activities of everyday living are essential to maintain independent functional mobility and are determinants of quality of life [1] and risk of falls [2] in elderly and impaired populations (e.g., stroke, multiple sclerosis, cerebral palsy, and Parkinson’s disease).

In a clinical setting, the monitoring, treatment, and evaluation of gait and balance deficits currently rely on intermittent use of standardized outcome tests; for example, tests of walking speed, walking distance, balance confidence, and the ability to hold static postures or complete dynamic movements. Many of these tests are scored using a single performance-based measure, whereas others are qualitative and may be subjective in that they rely on patient self-reports and therapist observations [3]. These measures, though effective in maintaining clinical integrity, lack the resolution needed to detect subtle, impairment-based changes occurring during the recovery process. Furthermore, these methods are subject to inter- and intra-observer variability [4], making it difficult to design person-specific, progressive therapeutic strategies to improve deficits. Automated systems, computing clinically-relevant measures from real-time data, would enable therapists and physicians to continuously track gait and balance objectively and at a higher resolution than currently possible. Such an approach would likely help clinicians make informed decisions about early interventions, treatment efficacy, and patient recovery progress.

Wearable sensors, such as inertial measurement units (IMUs), are promising tools to augment the current clinical tests of gait and balance. This technology provides continuous, objective, and high-resolution movement data that may better quantify test performance. These sensors are also relatively inexpensive, easy to use, lightweight, and unobtrusive compared with specialized laboratory equipment (i.e., force plates, infrared motion capture systems). It is not surprising then that there exists an extensive body of work regarding algorithm development for human motion analysis using IMUs. For instance, there are multiple systematic reviews that summarize studies of inertial sensors to estimate gait kinematic and kinetics [5], gait cycle segmentation by signal processing [6] or machine learning [7], and postural stability metrics [8]. Despite continued interest in wearable technology, there is still a lack of research that validates or implements sensor data in actual clinical practice. An exception is the work by Bergamini et al. [9], in which multiple inertial sensors were used to measure gait stability during a 10 m Walk Test (10MWT) in subacute stroke patients. Importantly, the sensor data were able to discriminate between different levels of walking ability as well as fall risk. In another example, the authors of [10] explored age-related changes of gait and balance using IMUs at multiple locations during a similar, though nonstandardized, Instrumented Stand and Walk test (iSAW). They found a linear deterioration of postural sway and gait with age for some features of sensor data, and alternative patterns for other features (e.g., deterioration after the 6th or 7th decade or no change throughout age span). The next logical step in this line of investigation is to examine sensor data collected during multiple standardized gait and balance tests, which are common tools used to evaluate aging or impaired individuals.

For a realistic implementation of wearable technology in the clinical setting, limiting the number of sensor devices is critical to day-to-day usability and field deployment. To minimize the temporal, physical, and cognitive burdens on clinicians, it is highly desirable to have the fewest number of devices to assess performance as possible, particularly for impaired populations. Various algorithms have been developed for a single IMU on the lower back (approximate location of center of mass) to quantify gait and balance. To augment measures currently used in the clinical setting, we propose a “clinical meta-feature extraction” (CMFE) process, which we define as a comprehensive combination of algorithms to extract quantitative features of gait and balance. The CMFE process is intended to consolidate previously developed signal processing approaches to robustly quantify gait and balance using IMU signal features during multiple standardized clinical tests.

Using CMFE, we sought to develop a normative dataset of gait and balance features from healthy, age-ranged individuals using a single inertial sensor on the lower back. We selected the BioStampRC sensor (MC10 Inc.; Lexington, MA, USA), which is a flexible, wireless, multimodal, research-grade device. This device was chosen for its low profile and flexible mechanical properties, as well as its ability to collect multiple sensing modalities (e.g., triaxial accelerometer and gyroscope data on a single device). Bilateral shank placement of the BioStampRC was previously validated against an activity monitor for counting steps and temporal measures of the gait cycle [11]. For a single device on the lower back, metrics from the BioStampRC have also been validated during standing balance against force plate data [12]. In the CMFE approach, we combined previous algorithms to extract kinematic movement estimates and descriptive signal characteristics in the time and frequency domains. These algorithms were selected for their relevance to the sensor location at the lower back, the clinical tests of interest, and previously demonstrated accuracy. The feature set included spatiotemporal gait kinematics (e.g., stance time and step length), which rely on accurate detection of gait cycle events such as heel strike and toe-off. Because of the novel combination of algorithms in CMFE for identifying gait cycle events, we also validated the subset of spatiotemporal gait features against gold standard measures.

The objectives of this study are threefold: (1) to validate sensor-derived spatiotemporal gait kinematics (based on the detection of gait cycle events) against gold standard measures to assess accuracy and bias, (2) to implement the CMFE approach to compute sensor-derived features of gait and balance during common clinical outcome measures for age-ranged healthy individuals, and (3) to quantify the effect of age and phenotype characteristics (sex, height, and weight) on these sensor-derived features. This approach lays a foundation to monitor high-resolution gait and balance measures in different impaired populations. As a proof of concept, we also applied CMFE to compute sensor-derived features for a single individual with stroke and compared their outcome data to the healthy group.

## 2. Materials and Methods

### 2.1. Participants

Fifty-one healthy adults participated in the study (N = 51; age range: 20 to 70). Two subjects were excluded from analysis due to issues with the sensor battery, resulting in 49 total subjects who served as a basis for three different age groups (Table 1). These participants had no known musculoskeletal or neurological issues. 

In addition, one 57-year-old male with a right-side pario-occipital and cerebellar hemorrhagic stroke participated while undergoing inpatient rehabilitation at the Shirley Ryan AbilityLab (Chicago, IL). The patient was 42 days post-stroke and presented with left-side hemiparesis. He was discharged from the hospital to his home the following day.

All individuals provided written informed consent before participation. The study was approved by the Institutional Review Board of Northwestern University (Chicago, IL, USA) in accordance with federal regulations, university policies, and ethical standards regarding research on human subjects.

### 2.2. Protocol and Data Collection

Participants performed a sequence of three tests based on common clinical outcome measures in random order:

1. The 10-m walk test (10MWT) of gait speed, with three trials each at a self-selected velocity (SSV) and fast velocity (FV). Increasing gait speed has been correlated with a higher quality of life [1] and community mobility [13]. The traditional clinical outcome of the 10MWT is average walking speed in the SSV and FV conditions. Participants walked over an instrumented walkway (GAITRite; CIR Systems, Inc., Franklin, NJ, USA) during this test, which was used as the gold standard for validating spatiotemporal gait characteristics computed from sensor data.
2. Static postural stability condition of the Berg Balance Scale (BBS), including standing unsupported with feet apart (SU), standing with eyes closed (SEC), standing with feet together (SFT), (d) standing in tandem stance (ST) with their nondominant (or paretic) leg behind, and standing on one leg (SOL) on their nondominant (or paretic) leg. This test assesses functional balance and is associated with risk of falling [2]. A trained clinician scores each item on a 5-point ordinal scale, ranging from 0 (lowest function) to 4 (highest function). The traditional clinical outcome of the BBS is the total score.
3. Timed Up and Go (TUG) test of functional mobility, with two trials collected. This test assesses functional mobility and is used to predict the risk of falls [14]. Participants began seated in a chair, rose to a standing position without use of their hands (Sit-to-Stand), walked 3 m (Walk), turned 180 degrees (Turn 1), walked 3 m back to the chair (Walk), turned 180° (Turn 2), and sat down in the chair without use of their hands (Stand-to-Sit). The traditional clinical outcome of the TUG is the total time required to complete the test.

To validate step count estimates, participants also performed four naturalistic walking trials in a circuit at a self-selected velocity. The circuit required approximately 91 m (300 ft) of walking, including straight walking, walking through three open doorways, and turning corners (two right turns, two left turns). Visual step count was recorded as the gold standard for validating step count computed from sensor data, obtained from a researcher walking behind the participant and clicking a tally-counter each time the participant’s foot impacted the ground. The individual with stroke performed a similar, abbreviated circuit, 33.5 m (110 ft) in length, on the inpatient hospital floor (one open doorway, one right turn, and one left turn).

### 2.3. Sensor Technology

**Figure 1:**
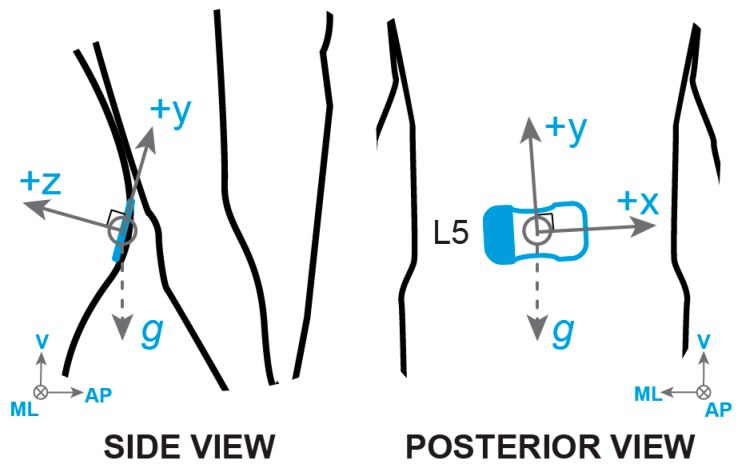
The BioStampRC sensor was secured to the skin at the L5 vertebra with adhesive film, aligned with the local coordinate system of the vertebra. The sensor recorded triaxial accelerometer and gyroscope signals. Positive and negative axes of the sensor were pre-defined by the BioStampRC and later aligned with the true (global) coordinate system for anteroposterior (AP), mediolateral (ML), and vertical (V) directions.

Participants wore a skin-mounted IMU (BioStampRC; MC10, Inc., Cambridge, MA, USA; dimensions: 65 × 35 × 3 mm, weight: 7 g) positioned on the fifth lumbar vertebra (L5), approximating the location of the body center of mass (CoM). The sensor was attached to the skin with an overlying layer of transparent adhesive film (Tegaderm; 3M, St. Paul, MN, USA). The BioStampRC collected triaxial acceleration (sensitivity ±4 g) and triaxial angular velocity (sensitivity ±2000°/s) at 31.25 Hz. Sensor axes were aligned with the local coordinate system of the L5 vertebra (Figure 1). A Samsung Galaxy tablet running the proprietary BioStampRC application was used to collect the sensor data and annotate the beginning and end of each trial/condition during the clinical tests.

De-identified sensor data were uploaded to the MC10 BioStampRC Cloud and then downloaded to a HIPAA-compliant (Health Insurance Portability and Accountability Act of 1996) secure server. Data processing and analysis were implemented in MATLAB 2017a (MathWorks, Natick, MA, USA).

### 2.4. Data Exclusions

Two individuals were excluded from certain clinical tests. One participant was excluded from analysis of the 10MWT in the FV condition because of a particularly high walking velocity (2.75 m/s); in this case, the sensor sampling rate was unable to capture the underlying time and frequency components needed to estimate the foot gait events. Additionally, one subject was excluded from the TUG analysis because of additional noise in the signals, likely due to poor sensor adhesion (i.e., from sweat or prolonged wear time of Tegaderm) and the resulting movement artifacts, which made it difficult to identify phases of the TUG. For the validating step count from the naturalistic walking bouts, three trials from a single subject were excluded due to a lack of visual step count to use as the gold standard.

### 2.5. Data Analysis

#### 2.5.1. Clinical Meta-Feature Extraction

The CMFE process involved extracting a wide-ranging set of sensor features from the clinical tests using the following process. First, the accelerometer signals were transformed to a horizontal–vertical coordinate system to correct for slight variations in sensor placement and so that the triaxial signals corresponded to dynamic accelerations in three true anatomical directions: anteroposterior (AP), mediolateral (ML), and vertical (V). This was done using the approach reported in [15], projecting the raw measured accelerations $a_{x}$, $a_{y}$, and $a_{z}$ to the anatomical planes and removing the static vertical acceleration due to gravity (1*g*). The true AP, ML, and V were estimated using a provisional vertical acceleration ${\hat{a}}_{V}$ and the following set of equations, where all values are normalized to gravity:

(1a) $${\hat{a}}_{V}=\;a_{z}\sin\theta_{z}+a_{y}\cos\theta_{z}$$

(1b) $$a_{\mathrm{AP}}=-a_{z}\cos\theta_{z}+a_{y}\sin\theta_{z}$$

(1c) $$a_{\mathrm{ML}}=-a_{x}\cos\theta_{x}+{\hat{a}}_{V}\sin\theta_{x}$$

(1d) $$a_{V}=a_{x}\sin\theta_{x}+{\hat{a}}_{V}\cos\theta_{x}-1.$$

here, $\theta_{x}$ and $\theta_{z}$ are the angles between the true horizontal (ML) plane and the IMU-fixed x- and z-axes, respectively, with positive rotation being upwards from the horizontal plane. These angles were computed using the mean acceleration $\bar{a}$ in that direction, based on the approximations $\sin\theta_{x}\approx {\bar{a}}_{x}$ and $\sin\theta_{z}\approx {\bar{a}}_{z}$ for large *n* [15]. Finally, all accelerations were converted to m/s^2^.

For walking-related tests (10MWT, TUG walking phase, and naturalistic walking bouts for step count), accelerometer signals were filtered using a fourth-order Butterworth low-pass filter at 10 Hz [16] to obtain preprocessed accelerations, $a_{\mathrm{AP}}\prime$, $a_{\mathrm{ML}}\prime$, and $a_{V}\prime$.

**Figure 2:**
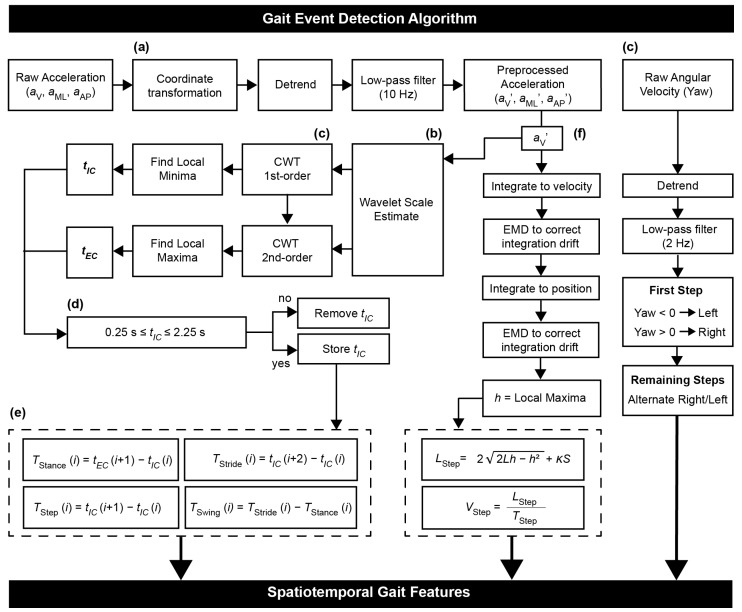
Flowchart to estimate spatiotemporal gait features, combining methodologies from (**a**) [15,16], (**b**) [16,23], (**c**) [17], (**d**) [18], (**e**) [16], and (**f**) [16,20]. $a_{V}$, $a_{\mathrm{ML}}$, $a_{\mathrm{AP}}$ = acceleration in vertical, mediolateral, and anterposterior directions, respectively. CWT = continuous wavelet transform; $t_{\mathrm{IC}}$ = times of initial contact; $t_{\mathrm{EC}}$ = times of end contact; *i* = index of gait cycle; $T_{\mathrm{Stance}}$ = stance time; $T_{\mathrm{Stride}}$ = stride time; $T_{\mathrm{Step}}$ = step time; $T_{\mathrm{Swing}}$ = swing time; EMD = empirical mode decomposition; *h* = vertical displacement of CoM; *L* = distance from sensor (approximately located at CoM) to ground during upright standing; K = optimization constant; $L_{\mathrm{Step}}$ = step length; $V_{\mathrm{Step}}$ = step velocity.

*Gait Event Detection Algorithm*: Gait events were detected using the flowchart in Figure 2. Foot contact events were estimated using a continuous wavelet transform approach (CWT) on the preprocessed vertical acceleration $a_{V}\prime$ [17]. This algorithm uses two wavelets—Gaussian and Mexican Hat—to detect initial contact (IC) and end contact (EC) respectively (function cwt in MATLAB). To determine the scale for each wavelet, a nonlinear frequency-scale relationship was implemented [16]. First, the acceleration signal was integrated and differentiated with respect to CWT using a Gaussian wavelet (gaus1), and the resulting local minima were identified as IC events. The signal was again differentiated using the Mexican Hat wavelet (gaus2), and the resulting local maxima were identified as EC events. Only peaks with a magnitude > 20% of the mean of all peaks were considered for EC detection. IC events were assumed to pair with the subsequent EC event, and additional (false) ICs were removed if they occurred within 0.25 s or outside 2.25 s of the previous IC [18]. Finally, the angular velocity about the vertical axis, also known as yaw, was filtered using a fourth-order low-pass Butterworth filter at 2 Hz to designate right and left leg gait events.

Temporal gait parameters for a gait cycle *i* were estimated as follows [16],

(2a) $$T_{\mathrm{Stance}}\left(i\right)=t_{\mathrm{EC}}\left(i+1\right)-t_{\mathrm{IC}}\left(i\right),$$

(2b) $$T_{\mathrm{Stride}}\left(i\right)=t_{\mathrm{IC}}\left(i+2\right)-t_{\mathrm{IC}}\left(i\right),$$

(2c) $$T_{\mathrm{Step}}\left(i\right)=t_{\mathrm{IC}}\left(i+1\right)-t_{\mathrm{IC}}\left(i\right),$$

(2d) $$T_{\mathrm{Swing}}\left(i\right)=T_{\mathrm{Stride}}\left(i\right)-T_{\mathrm{Stance}}\left(i\right),$$

where $T_{\mathrm{Stance}}$, $T_{\mathrm{Stride}}$, $T_{\mathrm{Step}}$, and $T_{\mathrm{Swing}}$ are the stance time, stride time, step time, and swing time, respectively, and $t_{\mathrm{EC}}$ and $t_{\mathrm{IC}}$ are the times of end contact and initial contact, respectively. Step count was defined as the number of initial contact events identified.

*Step Length Estimation Algorithm:* Step length was estimated using a modified inverted pendulum model. During gait, the CoM undergoes changes in height, which is used to estimate the step length ${\hat{L}}_{\mathrm{Step}}$ [19]:

(3) $${\hat{L}}_{\mathrm{Step}}\left(i\right)=2\sqrt{2Lh-h^{2}}$$

where L is the pendulum length (distance from the lower back sensor to the ground) and h is the change in height obtained by double integration of $a_{V}$. A constant offset is added to improve this estimate and compute a final step length $L_{\mathrm{Step}}$ [20]:

(4a) $$K={\left(S^{T}S\right)}^{-1}S^{T}\left(L_{\mathrm{Step}}^{*}-{\hat{L}}_{\mathrm{Step}}\right)$$

(4b) $$L_{\mathrm{Step}}\left(i\right)=2\sqrt{2Lh-h^{2}}+KS,$$

where S is the participant’s shoe size (vector of shoe sizes for all participants in Equation (4a)), K is an optimum proportional constant used for all participants, and $L_{\mathrm{Step}}^{*}$ is a vector array of the actual step length obtained from the gold standard (GAITRite) during the 10MWT. Because step length increases with walking speed, two constants were computed for the two velocity conditions for the healthy participants: K = 1.13 for SSV and K = 1.50 for FV. Separate K constants were computed for the stroke participant: K = −0.25 for SSV and K = −0.11 for FV. Without the KS correction term, ${\hat{L}}_{\mathrm{Step}}$ generally underestimated the actual step length for the healthy participants and overestimated actual step length for the stroke participant.

The integration drift of $a_{V}$ was removed by Empirical Mode Decomposition (EMD) [20,21]. First, the vertical velocity $v_{V}$ is obtained by integrating $a_{V}$ and then decomposed into Intrinsic Mode Functions (IMFs). Each IMF represents a component of the original $v_{V}$, from high-frequency to low-frequency components. To reconstruct $v_{V}$ without the integration drift, specific IMFs were selected using the Hurst exponent, which is a measure of predictability of a time series [22]. IMF components were visually inspected for the presence of trends to determine a quantitative cutoff of the Hurst exponent. Components with Hurst exponents > 0.8 were removed from the signal. The same process was applied when integrating $v_{V}$ to obtain a reconstructed version of the CoM vertical displacement h without drift.

*Static Postural Balance Algorithm:* The frequency-domain features (Table 2) were estimated using the fast Fourier transform (function “fft” in MATLAB). Time-domain features were estimated from the acceleration, as well as its differentiated (jerk) and integrated (velocity) signals. Finally, the ellipse features (Table 2) were obtained by computing the eigenvalues and eigenvectors of the covariance matrix of the acceleration signals in AP and ML planes [24].

**Figure 3:**
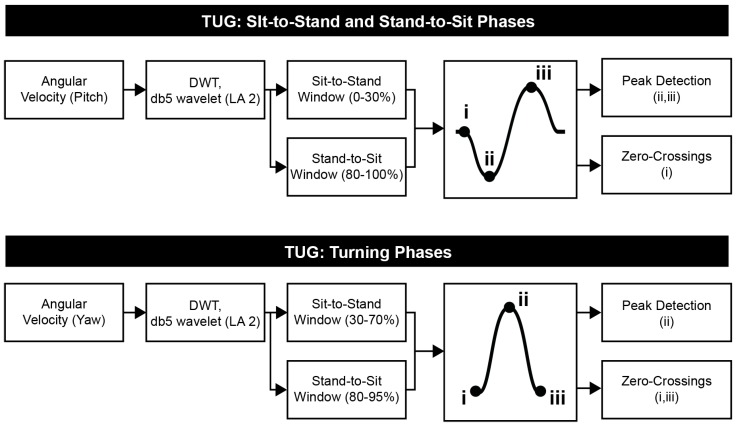
Flowchart for Timed Up and Go (TUG) phase detection (sit/stand transitions and turning). DWT = discrete wavelet transform; db5 = Daubechies 5; LA = level of approximation.

TUG Phase Detection Algorithm: This algorithm was developed to detect four main phases in the TUG: rising from a chair (sit-to-stand), walking, turning, and sitting down (stand-to-sit) [25,26,27]. Sit-to-Stand and Stand-to-Sit phases were estimated by a reconstruction of the pitch signal after using a discrete wavelet approach with a Daubechies mother wavelet (db5) and an approximation level 5 (5A). Two turning phases were identified under the same approach but using the yaw signal and an approximation level 2 (2A). Finally, the gait event detection algorithm described above was used to identify the walking phase, walking features, and step counts in each turn. A flowchart of the TUG Phase Detection Algorithm is given in Figure 3.

#### 2.5.2. Features Summary

A total of 184 features were calculated from the acceleration and angular velocity signals during the clinical tests and naturalistic walking bouts, summarized in Table 2. Of these, six features derived from gait event detection—stance time, swing time, step time, step length, step velocity, and step count—were first validated against gold standard measures.

**Figure 4:**
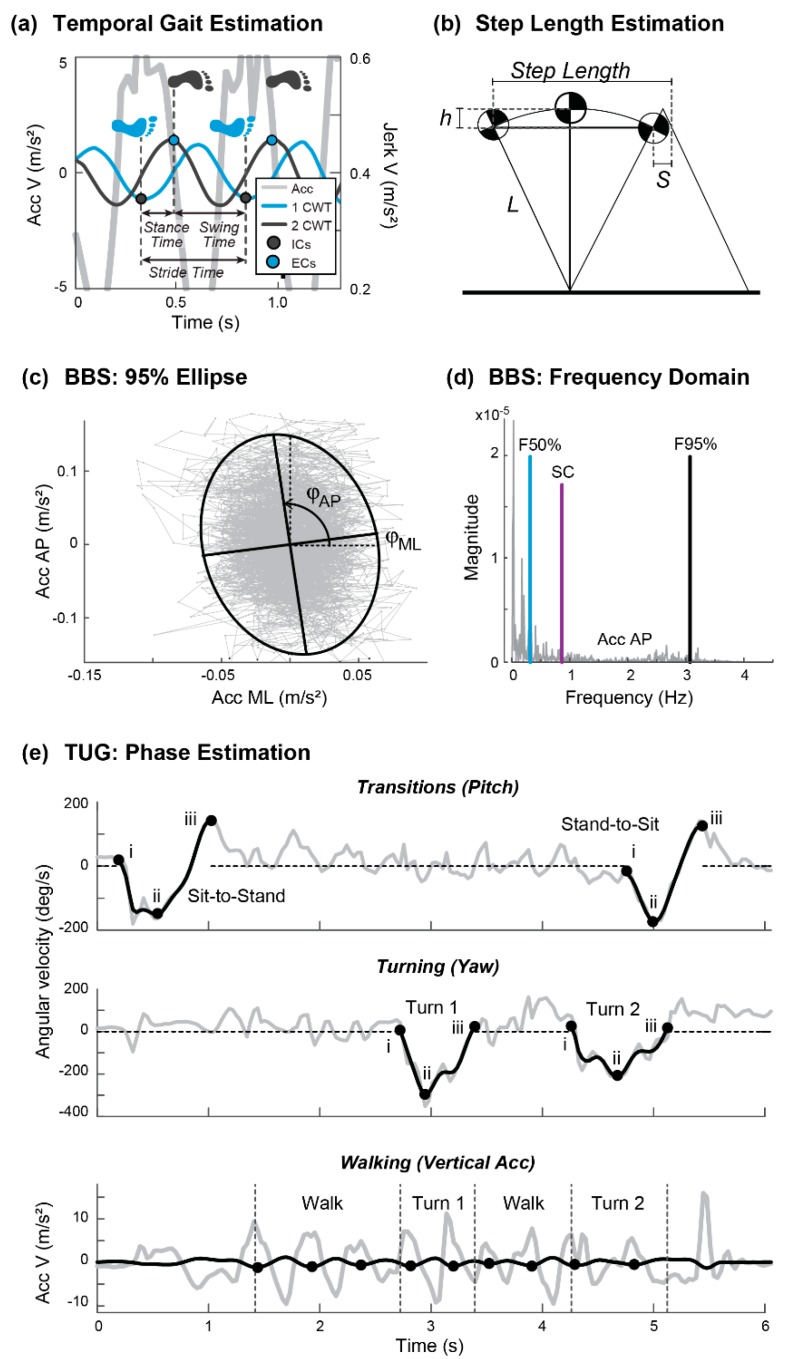
Examples of features estimated from the different clinical tests. (**a**) Temporal gait estimation by the CWT method. (**b**) Step length estimation by the inverted pendulum model. (**c**) 95% Ellipse area, axis, and angles. (**d**) Frequency domain measures from the Berg Balance Scale (BBS) (F50%, F95%, and SC). (**e**) Phase estimation in the Timed Up and Go (TUG) by the DWT method.

Examples of features and activity segmentation estimated from these algorithms are shown in Figure 4 for the clinical tests of gait and balance.

### 2.6. Statistical Analysis

Statistical analysis was performed using SPSS v25 (IBM, Armonk, NY, USA). Bland–Altman plots were used to express the error between the sensor system and the corresponding gold standard system (i.e., MC10 vs. GAITRite; MC10 vs. visual step count). Absolute agreement between the two systems was evaluated using intraclass correlations (ICC) and limits of agreement (LoA). Relative agreement between the systems was determined using Pearson’s correlation coefficient (*r*). Classification for ICC were considered as excellent (values greater than 0.9), good (between 0.75 and 0.9), moderate (between 0.5 and 0.75), or poor (less than 0.5) [30].

Overall, 183 of the 184 sensor-derived features were assessed across the three clinical tests for age effects (step count in the naturalistic walking bouts was used for validation only). Spatiotemporal gait features for the right and left legs were averaged for healthy participants, i.e., those who exhibited relatively symmetrical gait. Gait symmetry for the healthy participants was verified by comparing the empirical cumulative distribution for the left and right legs in each feature. Feature intercorrelations from each clinical test were explored using a correlation matrix and the Pearson correlation coefficients [10], to examine the presence or absence of relationships between gait and balance features.

The relationship between each feature and age was initially assessed using univariate correlations. Normality of the features was tested using D’Agostino–Pearson omnibus $K^{2}$ with significance level set to 0.05. Strength and direction of the correlations with age were measured with Pearson product–moment correlation for the normally distributed features, and Spearman’s rank order correlation for the non-normally distributed features. Partial correlations (r*) were performed to control for effects of weight and height. Correlations were considered non-negligible (that is, some association existed between the feature and age) for *r* values of 0.3 or greater [31]. 

Hierarchical multiple regression was performed to quantify the effect of age on features with significant, non-negligible univariate correlations (|*r*| ≥ 0.3, *p* < 0.05). The goal of these models was to determine whether adding age as a predictor variable significantly improves the proportion of explained variance ($R^{2}$) for the feature in question. Here, age was added as a predictor variable after adding the variables for weight, height, and sex, respectively [10], thereby testing the effect of age alone on a feature after controlling for other phenotype characteristics.

Finally, to identify the difference and level of resolution of the proposed features compared to current clinical outcome measures (total duration for the TUG, walking velocity for the 10MWT and therapist scores between 0 and 4 in the BBS), the same clinical outcome measures were tested for differences within clinical tests and among age groups using inferential statistics (two way ANOVAs with main effects of age and test condition, as well as their interaction).

## 3. Results

### 3.1. Validation of Spatiotemporal Gait Features

**Figure 5:**
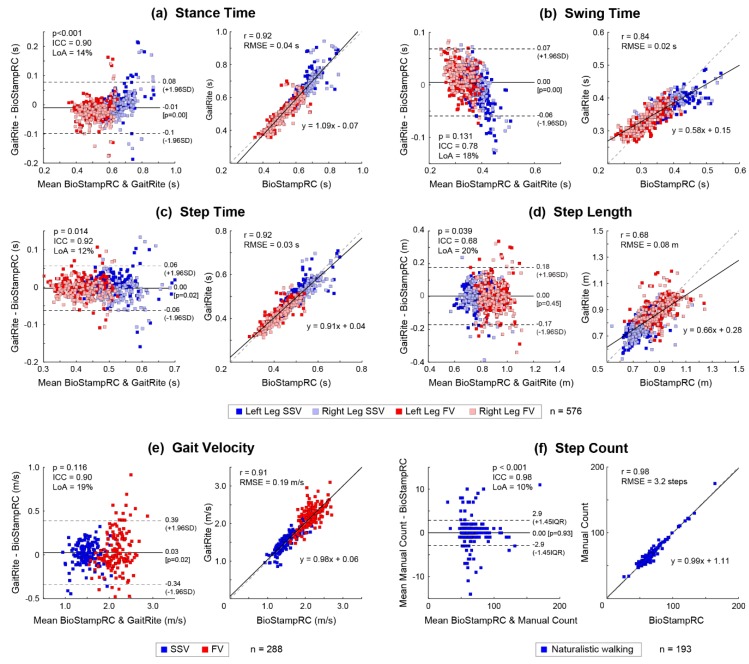
Bland–Altman and linear correlation plots between the BioStampRC and gold standard measures for spatiotemporal features of gait. (**a**) Stance time, (**b**) swing time, (**c**) step time, (**d**) step length, (**e**) gait velocity, and (**f**) step count. RMSE = Root-Mean-Squared Error; ICC = Intraclass Correlation; LoA = Limits of Agreement; *p*-value = D’Agostino–Pearson normality test.

Figure 5 summarizes validation of the spatiotemporal gait estimates. Bland–Altman plots show mean differences, ICC, and LoA (percentage and upper/lower bounds). Linear correlation plots show *p*-values for normality (D’Agostino–Pearson test), root-mean-squared error (RMSE), and linear equations between BioStampRC and gold standard measurements (GAITRite for spatiotemporal features and visual count for step count estimation).

There was excellent agreement between the gold standard measure and estimates from sensor data for most temporal gait features, including stance time, step time, and gait velocity (ICC > 0.90, LoA < 20%), as well as for step count (ICC = 0.98, LoA = 10%). There was good agreement for swing time (ICC = 0.78, LoA = 18%), with a notable trend of overestimating longer swing time. There was moderate agreement for step length (ICC = 0.68, LoA = 20%), with greater errors typically seen in the FV condition for larger steps.

### 3.2. Feature Independence between Clinical Tests

**Figure 6:**
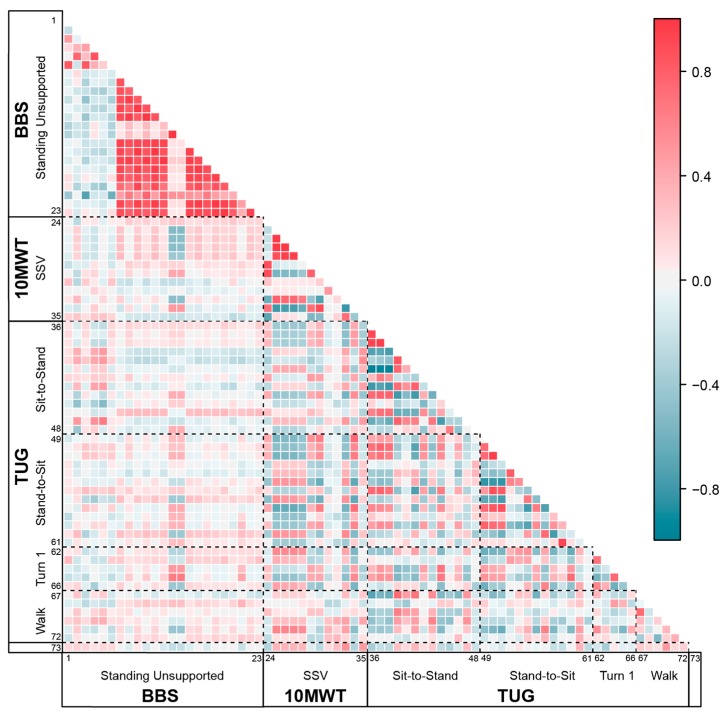
Correlation coefficients between sensor-derived features across clinical tests. BBS Standing Unsupported: 1–2. F50% (ML/AP), 3–4. F95% (ML/AP), 5–6. SC (AP/ML), 7–8. Max Acc (AP/ML), 9–10. Mean Acc (AP/ML), 11–12. RMS (AP/ML), 13–14. Ellipse Angle (AP/ML). 15. Ellipse Area, 16–17. Ellipse Axis (AP/ML), 18–19. Jerk (AP/ML), 20–21. SwayV (AP/ML), 22–23. SPathA (AP/ML). 10MWT SSV: 24. Mean Vertical Displacement, 25. Stance Time, 26. Step Time, 27. Stride Time, 28. Swing Time, 29. Step Length, 30. Power Frequency, 31. Stance Time Ratio, 32. Step Length Ratio, 33. Duration, 34. Mean Velocity, 35. N Steps. TUG Sit-to-Stand: 36–37. Range Pitch Vel (i–ii/ii–iii), 38. SD Pitch Vel (i–iii), 39. Mean Pitch Vel (i–iii), 40. Median Pitch Vel (i–iii), 41–42. Max Pitch Vel (i–ii/ii–iii), 43–44. Mean Pitch Acc (i–ii/ii–iii), 45. Mean Acc (AP), 46. SD Acc (AP), 47. Duration. 48. Median Acc (AP). TUG Stand-to-Sit: 49–50. Range Pitch Vel (i–ii/ii–iii), 51. SD Pitch Vel (i–iii), 52. Mean Pitch Vel (i–iii), 53. Median Pitch Vel (i–iii), 54–55. Max Pitch Vel (i–ii/ii–iii), 56–57. Mean Pitch Acc (i–ii/ii–iii), 58. Mean Acc (AP), 59. SD Acc (AP), 60. Duration, 61. Median Acc (AP). TUG Turn 1: 62. Duration, 63. N Steps, 64. Max Yaw Vel, 65–66. Mean Yaw Acc (i–ii/ii–iii). TUG Walk: 67–69. RMS (AP/ML/V), 70. N Steps, 71. Mean Step Time, 72. SD Step Time, 73. Velocity Difference (FV−SSV).

Figure 6 maps the Pearson product–moment correlation coefficients for the estimated features from one condition of each clinical test. Sensor-derived features were highly correlated within each clinical test, but clearly separable between the dynamic mobility (10MWT and TUG) and static balance (BBS) tests. This suggests that the features for each clinical test effectively represent different domains. Stronger correlations are seen between the 10MWT and TUG, which is expected as both tests include walking. In the BBS, time and frequency domain features were highly correlated within domains but showed almost no correlation between domains.

### 3.3. Correlation between Age and Sensor-Derived Features

Table A1, Table A2, Table A3, Table A4, Table A5, Table A6, Table A7 and Table A8 (see in Appendix A) describe how sensor-derived features across the different clinical tests are related to age. Statistically significant, non-negligible correlations with age (|r| ≥ 0.3, *p* < 0.05) were found in 28 out of 183 features across the three clinical tests. After adjusting for weight and height, this increased to 36 total features that were correlated with age. 

The strongest correlation with age was in the TUG second turn phase, for the yaw acceleration (i–ii) (r = −0.581, *p* ≤ 0.001; Table A8). This indicates that older participants rotated more slowly about their body midline in the first half of the second turn, before sitting. This particular finding illustrates the ability of sensorized motion to uncover behaviors that would be “invisible” to the typical clinician. Other moderate-to-strong correlations with age (|r| ≥ 0.3, *p* < 0.05) were found for the BBS: in SU (1/23 features), F95% ML; in SEC (1/23 features), SC ML; in SFT (1/23 features), F95% ML; in ST (1/23 features), F50% AP; and in SOL (5/23 features), maximum, mean, and RMS acceleration in the ML direction, AP ellipse axis, and ML sway velocity. For the 10MWT SSV (4/12), mean stance time, mean step length, mean velocity, and duration. For the 10MWT FV (5/12), mean step length, maximum power frequency, mean velocity, number of steps, and duration. For the TUG (17/42), in Sit-to-Stand phase, mean pitch velocity (i–iii), maximum pitch velocity and mean pitch acceleration (i–ii), and mean AP acceleration (i–iii); in Walking phase, RMS of AP acceleration, number of steps, and phase duration; in Turn 2 phase, maximum yaw velocity and mean yaw acceleration (ii–iii), and phase duration; finally, in the Stand-to-Sit phase, range and maximum pitch velocity (i–ii), range of pitch velocity and mean pitch acceleration (ii–iii), standard deviation of pitch velocity (i–iii), and mean and standard deviation of AP acceleration. As with the TUG, these results illustrate the power of quantifying motion as subtle differences between groups are easily determined.

### 3.4. Hierarchical Multivariate Regression for Age Effects in Sensor-Derived Features

Hierarchical multivariate regression was performed for the 36 features that had significant, non-negligible, independent correlations with age (|r| ≥ 0.3; *p* < 0.05) after correcting for weight and height (Table 3). This approach quantifies the relative effects of age, sex, height, and weight on the features of interest, and assesses the effect of age while controlling for these phenotype variables. The features included totaled nine in the BBS, nine in the 10MWT, and 18 in the TUG. Introducing age as a variable in the model significantly increased the amount of explained variance for all features, by 6.9–32.5%. From these 36 features, weight was also a significant predictor for F95% ML in the SU balance task, and for the Turn 2 duration in the TUG. Height was also a significant predictor for mean stance time in 10MWT-SSV. Sex was also a significant predictor for ML sway velocity in the SOL balance task, as well as mean step length and number of steps in 10MWT-FV. For the rest of the features, age was the only significant predictor.

### 3.5. Differences between Age Groups and Stroke Rehabilitation Participant

**Figure 7:**
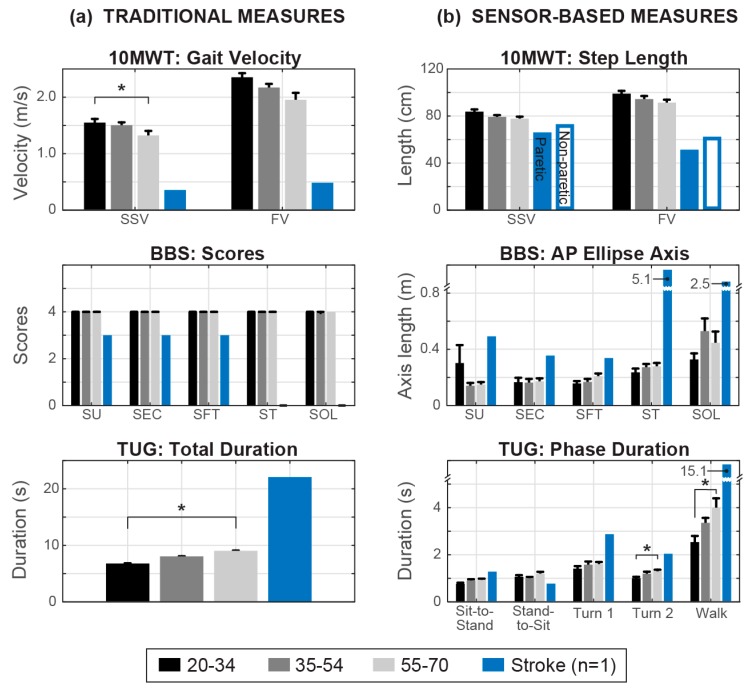
Clinical outcome comparison between (**a**) traditional measures and (**b**) sample features estimated by the sensor-derived approach in three age groups, as well as in a single stroke patient (42 days post-stroke, blue bar). SSV = Self-Selected Velocity; FV = Fast Velocity; SU = Standing Unsupported; SEC = Standing Eyes Closed; SFT = Standing Feet Together; ST = Standing Tandem stance; SOL = Standing on One Leg.

An example comparison of the clinical test scores and sensor-derived features is shown in Figure 7 for each test. A single sensor-derived feature was chosen from each test to illustrate differences between age groups and/or between these healthy participant groups and an individual with stroke.

The 10MWT is scored based on gait velocity, which decreased with age in both SSV and FV conditions (Figure 7a, top). There were main effects of age group (*p* = 0.003) and speed condition (*p* < 0.001) on gait velocity (*p* < 0.001), with no interaction effect (*p* = 0.95). Post hoc tests showed that the 55–70 age group had significantly lower gait velocities than the 20–34 age group (*p* = 0.001). Gait velocity for the stroke participant in SSV and FV was notably lower, at 0.36 and 0.48 m/s, respectively. For the sensor-derived feature of step length, mean values also decreased with age in both conditions (Figure 7b, top). There were main effects of age group (*p* = 0.014) and speed condition (*p* < 0.001) on step length, with no interaction effect (*p* = 0.91). Post hoc tests showed that the 55–70 age group had significantly shorter step length than the 20–34 age group (*p* = 0.006). The stroke participant took longer steps on his non-paretic side than his paretic side in both speed conditions, with shorter steps on average compared to healthy controls.

The BBS tasks are scored on a discrete scale of 0 to 4 by a therapist, for an individual’s ability to complete the task safely and for the required amount of time. All healthy participants received a perfect score on the static standing conditions, except one individual in the 34 to 54 age range who scored a 3 for Standing on One Leg because they could not hold the position for a full 10 seconds (Figure 7a, middle). The stroke participant scored a 3 on SU, SEC and SFT (able to complete under supervision) and a 0 on ST and SOL (loss of balance on attempt). The sensor approach provides continuous metrics to characterize performance, whereas specific differences between the balance conditions and age groups are seen in the length of the 95% ellipse anteroposterior axis computed from sensor data (Figure 7b, middle). Generally, AP axis length increased with the difficulty of the condition, meaning that individuals had greater acceleration in the forward–backward direction as balance was more challenged. There was a main effect of condition (*p* < 0.001) but not age group (*p* = 0.46) on AP axis length, and no significant interaction (*p* = 0.51). AP axis length for the stroke participant was greater in all conditions.

Finally, the TUG is scored as a time to complete all five phases of the test, which increased with age (Figure 7a, bottom). There was a main effect of age group on total TUG duration (*p* = 0.019), for which the 55–70 age group had significantly longer durations than the 20–34 age group (*p* = 0.007). The stroke participant completed the TUG in 22.06 s. Our CMFE approach can distinguish durations of each phase of the TUG to determine in which phase an individual moves faster or slower (Figure 7b, bottom). There were main effects of age group (*p* < 0.001) and phase (*p* < 0.001) on phase durations, as well as an interaction effect (*p* = 0.002). Post hoc tests showed that the 50–70 age group had a longer duration for the second turn (*p* = 0.041) and for the walking (*p* = 0.006) phase than the 20–34 age group. The duration of each phase for the stroke participant was sit-to-stand 1.25 s, stand-to-sit 0.74 s (uncontrolled descent), first turn 2.84 s, second turn 2.00 s, and walk 15.23 s. 

## 4. Discussion

In this study, we estimated features from different clinical tests performed in the rehabilitation setting using a novel combination of algorithms and data from a single IMU placed on the lower back. The end goal is to augment the information that can be obtained from current clinical tests, by automatically computing high-resolution measures of gait and balance.

The first objective was to validate a subset of sensor-derived spatiotemporal gait features against gold standard measures (GAITRite and visual step count) to explain systematic differences between the two systems. Temporal gait parameters in the 10MWT demonstrated excellent agreement (mean step time, stance time, and gait velocity) or good agreement (swing time), similar to previous work [16,17]. Step length demonstrated moderate agreement, with estimation errors that increased with gait velocity (SSV vs. FV conditions). 

A potential explanation for the lower accuracy in step length is in the walking kinematics of our participants. When modeling gait as a rigid inverted pendulum, it is assumed that the distance between the point of contact and the CoM is constant and an ideal pendulum has an equal exchange between kinetic and gravitational potential energy. In this model, increasing gait velocity increases the vertical displacement of the center of mass, and consequentially produces a larger step length. However, most of the subjects exhibited only a small change in vertical displacement between the fast and self-selected velocity conditions (mean 0.48 ± 0.88 cm), which would result in underestimation of the step length for faster gait velocities. The virtual limb model proposed by the authors of [32] may explain participants’ behavior. In this model, a virtual limb (pendulum) compresses in the stance phase at higher velocities, thereby reducing vertical displacement of the center of mass, and enhancing elastic energy storage (i.e., in the muscles and tendons). Another possible source of error in step length estimates is from the double integration to obtain position of the CoM from acceleration signals, as integration results in error accumulation and a “drift” in the integrated signal. Though we attempted to remove drift via EMD methods, other drift removal techniques paired with sensors at the foot may improve estimates [33]. Future work will examine the main source of step length error by comparing vertical excursions estimated from the inertial sensor signal to that from motion capture data.

The second and third objectives were to compute a series of sensor-derived features during clinical outcome tests of gait and balance, and to examine the effect of age and phenotype on these features. This was achieved using our novel CMFE process, combining several previously published algorithms to obtain a large, multidimensional feature set. These features are by no means exhaustive, and additional features may also be sensitive to age. For example, joint angles or muscle activation patterns would further augment test outcomes via additional sensor types and locations. Alternative algorithms for gait event detection could also be considered to improve accuracy of spatiotemporal features, include signal processing using different filters (FIR or IR) [6], sensor fusion [34], or machine learning [7]. We selected specific algorithms for gait event detection in CMFE based on the minimal selected sensor parameters (e.g., sensing types and position on body) and reliability for the target population, but different sensor types, body placement, or computational approaches could be substituted when extracting clinically-relevant features during standardized outcome measures. 

The extracted features included 23 in each of the five static balance conditions of the BBS, 42 across all phases of the TUG, 13 in each condition of the 10MWT, and an additional feature in the difference between self-selected and fast gait velocity in the 10MWT. Of these 184 total features, 36 were significantly correlated with age. Hierarchical multivariate regression confirmed that age was the most consistent contributor to changes in these features. Specific findings regarding age-related features are discussed below for each clinical outcome test. 

### 4.1. BBS Static Balance Performance

Our findings confirmed that balance declines with age across the static standing conditions (SU, SEC, SFT, ST, and SOL). Following the pattern reported by the authors of [10], participants demonstrated increasing time-domain sway features with age (i.e., mean and maximum velocity, acceleration, and jerk) and decreasing frequency domain features in the ML plane (F95% for SU and SFT, and SC for SEC). 

Age alone was the most significant predictor of seven features in the BBS, including positive correlations with time domain features for standing on one leg (Max Acc, Mean Acc, and RMS in the ML plane, and Ellipse Axis in the AP plane) and negative correlations with frequency domain features in the SEC, SFT, and ST tasks. This can be interpreted as larger and slower postural corrections with age. Aging affects neural factors such as increased reaction times [35] and biomechanical factors such as muscle weakness [35], which would affect balance performance in a pattern consistent with our findings.

### 4.2. 10MWT Performance

Gait velocity computed from the sensor data was negatively correlated with age in both self-selected and fast walking conditions, confirming that sensors can effectively capture the reductions in walking speed that are well-documented with age [36]. Step length decreased with age, whereas stance time increased with age and height.

### 4.3. TUG Performance

In line with previous studies, the sit-to-stand and stand-to-sit phases in TUG exhibited the strongest correlations with age, related to the angular velocity (pitch signal) [25]. Age alone was the most significant predictor of 17 features in TUG, including positive correlations with the mean (i–iii) and maximum velocity and mean acceleration (i–ii) of the pitch signal in the sit-to-stand phase, as well as the RMS of AP acceleration in the walking phase. In the stand-to-sit phase, we also found negative correlations with the range of pitch velocity, maximum velocity and mean acceleration (i–ii) of the yaw signal, and the mean and standard deviation of the AP acceleration. 

Aging causes lower limb strength deficits (i.e., hip and knee flexion/extension and ankle dorsiflexion) [37]. Our findings suggest that older individuals rely more on trunk momentum to stand up from a sitting position. Specifically, they exhibit increased flexion of the trunk to translate the CoM to the base of support and subsequently extend the trunk via increasing the angular velocity (pitch) that contributes to the CoM vertical momentum [38,39]. Finally, the negative correlations in the second turn and in the sit-to-stand phases suggest a slower and more controlled turn and transition to sitting.

### 4.4. Strengths and Limitations

The strength of this study is its deployability, which is based on the simplicity of using only a single sensor to quantify the effect of age on gait and balance during well-established clinical outcomes and validating spatiotemporal gait features with the lowest sampling rate reported in the literature to our knowledge. We saw that sensor-based features extracted from these clinical tests could be grouped into separate domains to assess balance or gait and gait mobility. These features expand the traditional one-dimensional measures of the clinical outcome tests. As illustrated in Figure 7, these sensor-derived features mitigate floor/ceiling effects by distinguishing continuous differences between individuals and tasks. Finally, we demonstrate a proof-of-concept to implement this approach in stroke patients, illustrating how sensor-derived features from a single patient can be compared to normative data from healthy, age-ranged cohorts.

There are some limitations to this study that should be considered. First, the sample size for each age group was relatively small, and the maximum age of participants in the study was 70 in a fairly active cohort of older adults. This limits our ability to predict age-related changes in sensor data for an older or more general population. For instance, the univariate correlations of sensor-based features were relatively weak, with 35 features exhibiting a low correlation (|*r*| values 0.3 to 0.5) after controlling for weight and height, one feature exhibiting a moderate correlation (0.5 to 0.7), and no features exhibiting high (0.7 to 0.9) or very high correlations (0.9 to 1.0) [31]. Performance in BBS, 10MWT, and TUG continues to decline for individuals over the age of 70 [36], so extending the age range may capture a stronger relationship between sensor features and age. Similarly, for sensor-based features showing a non-negligible correlation with age (*|r*| ≥ 0.3, *p* < 0.05), the hierarchical multivariate regression model of age, gender, height, and weight yielded relatively low R^2^ values, ranging from 0.104 to 0.604. This suggests that the 4-variable model does not explain most of the variance in this data and would be insufficient to predict outcomes accurately. However, the significance of age or other phenotype characteristics as predictors indicates a relationship between these variables and the sensor-based features. Further research is required to determine additional predictors of these sensor-based features, as well as their clinical relevance to age or impairment.

It should also be noted that, in the current study, estimating step length from sensor data requires an optimization constant K that is derived from gold standard data (in this case, actual step length from a GAITRite instrumented mat), as described in Equation (4a). Without this constant and the correction term presented in Equation (4b), we observed that step length was generally underestimated for the healthy group and overestimated for the stroke participant. Thus, we believe it would be critical to compute new, robust K values for gait-impaired populations, to ensure that the procedure would generalize to new individuals without measuring actual step length each time.

Future work will incorporate IMUs at the lower limbs to improve the step length estimation, and to maintain accuracy in step detection and spatiotemporal kinematics for gait-impaired populations [40]. Although a single lower back IMU was generally sufficient for gait cycle segmentation for healthy or mildly impaired individuals, additional IMUs or magneto-inertial sensors placed more distally (closer to the point of foot impact) are likely necessary when patients exhibit more drastic functional impairments such as slow walking [41], dropped foot [42], shuffling, or non-alternating steps [43].

## 5. Conclusions

In summary, we validated spatiotemporal gait parameters and quantified age-related changes across well-established clinical outcome tests used to quantify gait, mobility, and balance by applying a single IMU to the lower back. We demonstrated that sensor-derived features can improve the resolution required to determine changes related to age and augment current clinical outcome measures. Our results suggest that TUG is a reliable test for the quantification of age-related differences. The clinical meta-feature extraction approach with a single inertial sensor was feasible for estimating temporal gait features, though less accurate for step length estimation using the inverted pendulum model.

Overall, this study lays a foundation for amassing clinically-relevant baseline features from a healthy population to evaluate recovery progression across different impaired populations (e.g., stroke, multiple sclerosis, etc.). We expect that this approach would allow clinicians and therapists to better distinguish individual differences when evaluating gait and balance in the laboratory or in the community, thereby paving the way for more data-driven diagnoses and treatment of mobility impairment.

## Figures and Tables

**Table 1:** Participant demographics with mean (SD).

Group	N	Age (years)	Height (cm)	Weight (kg)	Female	Male
Ages 20–34	14	26.4 (3.9)	173.0 (11.4)	71.5 (13.8)	6	8
Ages 35–54	19	43.7 (5.8)	169.9 (12.8)	79.2 (23.7)	11	8
Ages 55–70	16	61.8 (5.1)	169.7 (7.5)	73.1 (15.9)	8	8
Stroke	1	57	185.4	82.8	0	1

**Table 2:** Sensor features computed for each clinical test.

Test	Feature	Reference	Units	Definition
BBS	F50% (AP, ML)	[28,29]	Hz	Frequency accounting for 50% of total power of the signal
	F95% (AP, ML)	[28,29]	Hz	Frequency accounting for 95% of total power of the signal
	SC (AP, ML)	[28,29]	Hz	Spectral centroid (indicates center of mass of the spectrum)
	Max Acc (AP, ML)		m/s^2^	Maximum acceleration
	Mean Acc (AP, ML)		m/s^2^	Mean acceleration
	RMS (AP, ML)	[12,28]	m/s^2^	Root mean square of acceleration
	Ellipse Angles (AP, ML)	[12,29]	m/s^2^	Angles of 95% of ellipse orientation
	95% Ellipse Area		m^2^/s^4^	Area of 95% ellipse
	Ellipse Axis (AP, ML)	[29]	m/s^2^	Length of 95% ellipse axis
	Jerk (AP, ML)	[12]	m/s^3^	Smoothness of sway (time derivative of acceleration)
	SwayV (AP, ML)	[12,28]	m/s	Mean sway velocity
	SPathA (AP, ML)	[28]	m/s^2^	Total acceleration path
10MWT	Mean Vertical Displacement		m	Vertical displacement of the body Center of Mass (CoM)
	**Mean Stance Time (SSV, FV)**	**[16]**	**s**	**Length of time for which the foot is in contact with the ground**
	**Mean Step Time (SSV, FV)**	**[16]**	**s**	**Length of time between successive ICs of opposite feet**
	**Mean Stride Time (SSV, FV)**	**[16]**	**s**	**Length of time between successive ICs of the same foot**
	**Mean Swing Time (SSV, FV)**	**[16]**	**s**	**Length of time for which the foot is not in contact with the ground**
	**Mean Step Length (SSV, FV)**	**[16]**	**cm**	**Distance between successive ICs of opposite feet**
	Maximum Power Frequency (SSV, FV)		(m/s^2^)^2^/Hz	Maximum power from the power spectral density of vertical acceleration
	Stance Time Symmetry Ratio (SSV, FV)		unitless	Stance time ratio of right and left leg (temporal symmetry)
	Step Length Symmetry Ratio (SSV, FV)		unitless	Step length ratio of right and left leg (spatial symmetry)
	Duration (SSV, FV)		s	Time required to complete the test, averaged over three trials
	**Mean Velocity (SSV, FV)**	**[16]**	**m/s**	**Mean step velocity**
	N Steps (SSV, FV)		unitless	Number of steps taken
	Velocity Difference, FV–SSV		m/s	Difference in average walking velocity between SSV and FV modes
TUG—Sit to Stand, Stand to Sit	Range Pitch Vel (i–ii, ii–iii)^2^	[25]	°/s	Difference between the minimum and maximum values of angular velocity (pitch axis)
	SD Pitch Vel (i–iii)	[25]	°/s	Standard deviation of angular velocity (pitch axis)
	Mean Pitch Vel (i–iii)	[25]	°/s	Mean value of angular velocity (pitch axis)
	Median Pitch Vel (i–iii)	[25]	°/s	Median value of angular velocity (pitch axis)
	Max Pitch Vel (i–ii, ii–iii)	[25]	°/s	Maximum value of angular velocity (pitch axis)
	Mean Pitch Acc (i–ii, ii–iii)	[25]	°/s^2^	Average rate of change of angular velocity (angular acceleration, pitch axis)
	Mean Acc AP (i–iii)	[25]	m/s^2^	Mean phase value of AP acceleration
	SD Acc AP (i–iii)	[25]	m/s^2^	Standard deviation of AP acceleration
	Median Acc AP (i–iii)	[25]	m/s^2^	Median value of AP acceleration
	Duration (i–iii)	[25]	s	Time required to complete the phase
TUG—Turn 1, Turn 2	N Steps	[25]	unitless	Number of steps taken
	Max Yaw Vel	[25]	°/s	Maximum value of angular velocity magnitude (yaw axis)
	Mean Yaw Acc (i–ii, ii–iii)	[25]	°/s^2^	Average rate of change of angular velocity (angular acceleration, yaw axis)
	Duration	[25]	s	Time required to complete the turn phase
TUG—Walk 1 + Walk 2	RMS Acc (AP, ML, V)	[25]	m/s^2^	Root mean square of acceleration
	Mean Step Time	[25]	s	Mean step time over the two walking phases
	SD Step Time	[25]	s	Standard deviation of step time
	N Steps	[25]	unitless	Number of steps taken
	Duration	[25]	s	Time required to complete the walking phase
Naturalistic Walking	**N Steps**		**unitless**	**Number of steps taken**

AP = anteroposterior; ML = mediolateral; V = vertical; SSV = self-selected velocity; FV = fast velocity; i–ii = first stage of the movement; ii–iii = second stage of the movement. Features in bold were validated against gold standard measures (Section 3.1).

**Table 3:** Hierarchical multiple regression analysis from the features that showed non-negligible correlations with age (|*r*| ≥ 0.3, *p* < 0.05) after correcting for weight and height.

Feature	Model no.	Standardized Beta Coefficients				*R* ^2^	*R*^2^ Change	*F* Change	df	*p*
		Weight	Height	Sex	Age					
BBS-SU										
F95% ML	1	−0.246				0.061	0.061	3.03	1, 47	0.088
	2	**−0.429**	0.295			0.114	0.054	2.78	1, 46	0.10
	3	**−0.416**	0.347	−0.076		0.116	0.002	0.10	1, 45	0.75
	4	**−0.374**	0.305	−0.074	**−0.363**	0.247	0.131	7.62	1, 44	**0.008**
BBS-SEC										
SC ML	1	−0.100				0.010	0.010	0.48	1, 47	0.49
	2	−0.206	0.170			0.028	0.018	0.84	1, 46	0.37
	3	−0.184	0.251	−0.119		0.033	0.005	0.23	1, 45	0.63
	4	−0.143	0.209	−0.117	**−0.365**	0.165	0.132	6.95	1, 44	**0.012**
BBS-SFT										
F95% ML	1	−0.165				0.027	0.027	1.31	1, 47	0.26
	2	−0.212	0.077			0.031	0.004	0.17	1, 46	0.68
	3	−0.162	0.268	−0.281		0.058	0.028	1.31	1, 45	0.26
	4	−0.127	0.234	−0.279	**−0.301**	0.148	0.090	4.65	1, 44	**0.037**
BBS-ST										
F50% AP	1	0.022				<0.001	<0.001	0.02	1, 47	0.88
	2	−0.073	0.152			0.015	0.015	0.68	1, 46	0.42
	3	-0.071	0.158	−0.008		0.015	<0.001	0.001	1, 45	0.98
	4	−0.037	0.123	−0.006	**−0.300**	0.104	0.089	4.39	1, 44	**0.042**
BBS-SOL										
Max Acc ML	1	−0.059				0.003	0.003	0.16	1, 47	0.69
	2	−0.157	0.158			0.019	0.015	0.72	1, 46	0.40
	3	−0.092	0.405	−0.362		0.065	0.046	2.20	1, 45	0.15
	4	−0.131	0.445	−0.364	**0.347**	0.184	0.119	6.42	1, 44	**0.015**
Mean Acc ML	1	−0.120				0.014	0.014	0.69	1, 47	0.41
	2	−0.164	0.070			0.017	0.003	0.14	1, 46	0.71
	3	−0.098	0.321	−0.368		0.065	0.047	2.27	1, 45	0.14
	4	−0.140	0.363	−0.370	**0.376**	0.205	0.140	7.74	1, 44	**0.008**
RMS ML	1	−0.091				0.008	0.008	0.40	1, 47	0.53
	2	−0.159	0.109			0.016	0.007	0.34	1, 46	0.56
	3	−0.098	0.342	−0.341		0.056	0.041	1.94	1, 45	0.17
	4	−0.140	0.385	−0.343	**0.378**	0.198	0.142	7.78	1, 44	**0.008**
Ellipse Axis AP	1	−0.030				0.001	0.001	0.04	1, 47	0.84
	2	−0.110	0.128			0.011	0.010	0.47	1, 46	0.50
	3	−0.042	0.386	−0.377		0.061	0.050	2.38	1, 45	0.13
	4	−0.078	0.422	−0.379	**0.316**	0.160	0.099	5.19	1, 44	**0.028**
SwayV ML	1	−0.187				0.035	0.035	1.70	1, 47	0.20
	2	−0.192	0.009			0.035	<0.001	0.002	1, 46	0.96
	3	−0.109	0.322	−0.460		0.109	0.074	3.725	1, 45	0.060
	4	−0.146	0.359	**−0.462**	**0.321**	0.211	0.102	5.678	1, 44	**0.022**
10MWT-SSV										
Mean Stance Time	1	**0.373**				0.139	0.139	7.59	1, 47	**0.008**
	2	0.051	**0.521**			0.307	0.168	11.14	1, 46	**0.002**
	3	0.016	0.384	0.200		0.321	0.014	0.91	1, 45	0.35
	4	−0.022	**0.423**	0.206	**0.265**	0.390	0.069	4.97	1, 44	**0.031**
Mean Step Length	1	**0.388**				0.151	0.151	8.34	1, 47	**0.006**
	2	0.076	**0.506**			0.309	0.158	10.53	1, 46	**0.002**
	3	0.026	0.313	0.280		0.336	0.027	1.84	1, 45	0.18
	4	0.069	0.268	0.272	**−0.305**	0.427	0.092	7.03	1, 44	**0.011**
Duration	1	0.186				0.035	0.035	1.69	1, 47	0.20
	2	0.100	0.139			0.047	0.012	0.58	1, 46	0.45
	3	0.087	0.089	0.074		0.049	0.002	0.09	1, 45	0.77
	4	0.044	0.134	0.082	**0.306**	0.140	0.092	4.69	1, 44	**0.036**
Mean Velocity	1	−0.044				0.002	0.002	0.09	1, 47	0.77
	2	−0.009	−0.057			0.004	0.002	0.09	1, 46	0.76
	3	−0.010	−0.063	0.009		0.004	<0.001	0.001	1, 45	0.97
	4	0.044	−0.121	−0.001	**−0.388**	0.152	0.148	7.68	1, 44	**0.008**
10MWT-FV										
Mean Step Length	1	**0.516**				0.266	0.266	16.67	1, 47	**<0.001**
	2	0.221	**0.485**			0.414	0.148	11.38	1, 46	**0.002**
	3	0.122	0.097	**0.566**		0.527	0.113	10.50	1, 45	**0.002**
	4	0.160	0.055	**0.559**	**−0.281**	0.604	0.077	8.41	1, 44	**0.006**
Maximum Power Frequency	1	0.138				0.019	0.019	0.89	1, 47	0.35
	2	0.140	−0.004			0.019	<0.001	0.001	1, 46	0.98
	3	0.095	−0.179	0.255		0.042	0.023	1.05	1, 45	0.31
	4	0.145	−0.233	0.246	**−0.361**	0.170	0.128	6.61	1, 44	**0.014**
Duration	1	0.104				0.011	0.011	0.51	1, 47	0.48
	2	0.145	−0.067			0.014	0.003	0.13	1, 46	0.72
	3	0.152	−0.041	−0.038		0.014	0.001	0.02	1, 45	0.88
	4	0.107	0.008	−0.030	**0.328**	0.120	0.106	5.16	1, 44	**0.028**
Mean Velocity	1	0.155				0.024	0.024	1.14	1, 47	0.29
	2	0.110	0.074			0.028	0.003	0.16	1, 46	0.69
	3	0.083	−0.032	0.155		0.036	0.008	0.39	1, 45	0.54
	4	0.133	−0.086	0.146	**−0.367**	0.168	0.132	6.81	1, 44	**0.012**
N Steps	1	−0.259				0.067	0.067	3.31	1, 47	0.075
	2	0.022	−0.463			0.202	0.135	7.63	1, 46	**0.008**
	3	0.100	−0.162	**−0.439**		0.270	0.068	4.10	1, 45	**0.049**
	4	0.055	−0.114	**−0.431**	**0.327**	0.375	0.105	7.21	1, 44	**0.010**
TUG- SIT-TO-STAND										
Mean Pitch Vel (i–iii)	1	0.101				0.010	0.010	0.47	1, 47	0.50
	2	0.338	**−0.383**			0.100	0.090	4.51	1, 46	**0.039**
	3	0.240	−0.378	−0.007		0.100	<0.001	0.001	1, 45	0.98
	4	0.304	−0.344	−0.005	**0.295**	0.186	0.086	4.55	1, 44	**0.039**
Max Pitch Vel (i–ii)	1	0.227				0.052	0.052	2.51	1, 47	0.12
	2	**0.373**	−0.235			0.086	0.034	1.67	1, 46	0.20
	3	0.371	−0.243	0.012		0.086	<0.001	0.002	1, 45	0.961
	4	0.336	−0.210	0.014	**0.290**	0.169	0.083	4.29	1, 44	**0.044**
Mean Pitch Acc (i–ii)	1	0.036				0.001	0.001	0.06	1, 47	0.81
	2	0.201	−0.266			0.045	0.043	2.05	1, 46	0.16
	3	0.162	−0.411	0.214		0.061	0.016	0.75	1, 45	0.39
	4	0.116	−0.366	0.217	**0.385**	0.207	0.146	7.94	1, 44	**0.007**
Duration (i–iii)	1	0.091				0.008	0.008	0.39	1, 47	0.54
	2	0.165	−0.119			0.017	0.009	0.40	1, 46	0.53
	3	0.144	−0.197	0.115		0.022	0.005	0.21	1, 45	0.649
	4	0.103	−0.157	0.118	**0.344**	0.138	0.117	5.83	1, 44	**0.020**
TUG-WALK										
RMS Acc AP	1	0.118				0.014	0.014	0.65	1, 47	0.43
	2	0.272	−0.248			0.052	0.038	1.80	1, 46	0.19
	3	0.333	−0.023	−0.332		0.091	0.039	1.87	1, 45	0.18
	4	0.290	0.018	−0.329	**0.357**	0.217	0.126	6.94	1, 44	**0.012**
N Steps	1	0.024				0.001	0.001	0.03	1, 47	0.87
	2	−0.060	0.136			0.012	0.011	0.52	1, 46	0.48
	3	−0.057	0.148	−0.017		0.012	<0.001	0.005	1, 45	0.95
	4	−0.115	0.203	−0.013	**0.483**	0.243	0.231	13.10	1, 44	**0.001**
Duration	1	0.124				0.015	0.015	0.71	1, 47	0.40
	2	0.027	0.155			0.030	0.015	0.69	1, 46	0.41
	3	0.018	0.120	0.051		0.031	0.001	0.04	1, 45	0.84
	4	−0.040	0.176	0.055	**0.483**	0.262	0.231	13.48	1, 44	**0.001**
TUG-TURN 2										
Max Yaw Vel	1	−0.136				0.019	0.019	0.87	1, 47	0.36
	2	−0.167	0.049			0.020	0.001	0.07	1, 46	0.80
	3	−0.178	0.007	0.062		0.021	0.001	0.06	1, 45	0.80
	4	−0.124	−0.045	0.058	**−0.453**	0.224	0.203	11.23	1, 44	**0.002**
Mean Yaw Acc (i–ii)	1	−0.145				0.021	0.021	0.99	1, 47	0.33
	2	−0.051	−0.153			0.035	0.014	0.67	1, 46	0.42
	3	−0.061	−0.193	0.059		0.037	0.001	0.06	1, 45	0.81
	4	0.007	−0.259	0.055	**−0.573**	0.362	0.325	21.88	1, 44	**<0.001**
Mean Yaw Acc (ii–iii)	1	**0.300**				0.090	0.090	4.55	1, 47	**0.038**
	2	0.353	−0.086			0.094	0.005	0.23	1, 46	0.64
	3	0.331	−0.169	0.123		0.100	0.005	0.26	1, 45	0.61
	4	0.294	−0.135	0.126	**0.302**	0.190	0.090	4.79	1, 44	**0.034**
Duration	1	**0.459**				0.210	0.210	12.26	1, 47	**0.001**
	2	**0.460**	−0.001			0.210	<0.001	<0.001	1, 46	0.99
	3	**0.453**	−0.026	0.036		0.211	<0.001	0.03	1, 45	0.87
	4	**0.411**	0.014	0.039	**0.349**	0.331	0.121	7.75	1, 44	**0.008**
TUG-STAND-TO-SIT										
Range Pitch Vel (i–ii)	1	**−0.331**				0.109	0.109	5.64	1, 47	**0.022**
	2	−0.297	−0.054			0.111	0.002	0.09	1, 46	0.76
	3	−0.259	0.084	−0.204		0.126	0.015	0.74	1, 45	0.40
	4	−0.219	0.045	−0.207	**−0.339**	0.239	0.113	6.41	1, 44	**0.015**
Range Pitch Vel (ii–iii)	1	**−0.408**				0.166	0.166	9.16	1, 47	**0.004**
	2	**−0.388**	−0.031			0.167	0.001	0.03	1, 46	0.86
	3	−0.314	0.244	−0.406		0.225	0.058	3.30	1, 45	0.076
	4	−0.280	0.212	−0.408	**−0.279**	0.302	0.077	4.74	1, 44	**0.035**
SD Pitch Vel (i–iii)	1	**−0.401**				0.161	0.161	8.83	1, 47	**0.005**
	2	−0.333	−0.109			0.168	0.007	0.40	1, 46	0.53
	3	−0.272	0.119	−0.337		0.208	0.040	2.22	1, 45	0.14
	4	−0.235	0.083	−0.339	**−0.308**	0.302	0.094	5.80	1, 44	**0.020**
Mean Pitch Acc (i–ii)	1	0.196				0.038	0.038	1.84	1, 47	0.18
	2	0.101	0.154			0.053	0.015	0.69	1, 46	0.41
	3	0.038	−0.076	0.339		0.094	0.041	1.97	1, 45	0.17
	4	0.004	−0.043	0.341	**0.286**	0.174	0.081	4.21	1, 44	**0.046**
Mean Pitch Acc (ii–iii)	1	**−0.440**				0.194	0.194	11.04	1, 47	**0.002**
	2	−0.319	−0.195			0.217	0.023	1.35	1, 46	0.25
	3	−0.290	−0.088	−0.158		0.226	0.009	0.50	1, 45	0.48
	4	−0.245	−0.132	−0.161	**−0.376**	0.365	0.140	9.46	1, 44	**0.004**
Mean Acc AP (i–iii)	1	**−0.320**				0.102	0.102	5.25	1, 47	**0.027**
	2	−0.176	−0.233			0.136	0.033	1.74	1, 46	0.19
	3	−0.142	−0.109	−0.183		0.148	0.012	0.61	1, 45	0.44
	4	−0.097	−0.152	−0.186	**−0.372**	0.285	0.137	8.25	1, 44	**0.006**
SD Acc AP (i–iii)	1	−0.279				0.078	0.078	3.89	1, 47	0.055
	2	−0.233	−0.075			0.081	0.003	0.17	1, 46	0.68
	3	−0.174	0.143	−0.322		0.118	0.036	1.82	1, 45	0.19
	4	−0.136	0.107	−0.324	**−0.315**	0.216	0.098	5.39	1, 44	**0.025**

Bolded values indicate the predictor was significant (Standardized Beta Coefficients column) or that the regression model produced a significant F change (p column). Significance level was set to 0.05.

## Appendix A

**Table A1:** Correlation of Berg Balance Scale (BBS) (Standing Unsupported) features with age (N = 49).

Feature ^1^	Normality	Correlation with Age		Controlled for Weight and Height	
	*p*	*r*	*p*	*r* *	*p* *
*F50% AP*	0.001	−0.036	0.805	−0.110	0.463
*F50% ML*	<0.001	−0.146	0.316	0.001	0.994
F95% AP	0.062	−0.077	0.598	−0.058	0.697
*F95% ML*	0.009	**−0.426 ****	0.002	**−0.384 ****	0.008
SC AP	0.266	−0.134	0.359	−0.120	0.421
SC ML	0.188	−0.266	0.064	−0.253	0.086
*Max Acc AP*	<0.001	−0.005	0.974	0.066	0.659
*Max Acc ML*	<0.001	0.063	0.667	0.177	0.235
*Mean Acc AP*	<0.001	0.123	0.399	0.088	0.558
*Mean Acc ML*	<0.001	0.179	0.218	0.135	0.365
*RMS AP*	<0.001	0.125	0.391	0.063	0.672
*RMS ML*	<0.001	0.184	0.205	0.166	0.265
*Ellipse Angle AP*	<0.001	0.103	0.483	−0.187	0.209
*Ellipse Angle ML*	<0.001	0.198	0.173	0.248	0.092
*95% Ellipse Area*	<0.001	0.141	0.333	0.038	0.798
*Ellipse Axis AP*	<0.001	0.124	0.394	0.071	0.634
*Ellipse Axis ML*	<0.001	0.110	0.454	0.095	0.526
*Jerk AP*	<0.001	−0.012	0.933	−0.011	0.943
*Jerk ML*	<0.001	−0.105	0.473	−0.120	0.423
*SwayV AP*	<0.001	0.129	0.375	0.082	0.583
*SwayV ML*	<0.001	0.171	0.240	0.182	0.222
*SPathA AP*	<0.001	−0.015	0.916	−0.007	0.963
*SPathA ML*	<0.001	−0.099	0.496	−0.117	0.435

^1^ Features in italic were tested using the Spearman rank correlation; the remainder were tested with Pearson correlation. Bolded values indicate significant correlation: * *p* < 0.05, ** *p* < 0.01.

**Table A2:** Correlation of BBS (Standing Eyes Closed) features with age (N = 49).

Feature ^1^	Normality	Correlation with Age		Controlled for Weight and Height	
	*p*	*r*	*p*	*r* *	*p* *
*F50% AP*	0.010	0.023	0.899	−0.076	0.678
*F50% ML*	<0.001	−0.233	0.185	−0.308	0.087
*F95% AP*	<0.001	−0.214	0.255	−0.217	0.234
*F95% ML*	0.008	−0.145	0.412	−0.168	0.358
*SC AP*	<0.001	−0.115	0.516	−0.147	0.422
SC ML	0.305	**−0.378 ****	0.007	**−0.369 ***	0.011
*Max Acc AP*	<0.001	0.196	0.266	0.151	0.410
*Max Acc ML*	<0.001	0.072	0.687	0.039	0.833
*Mean Acc AP*	<0.001	0.107	0.548	0.174	0.341
*Mean Acc ML*	<0.001	0.151	0.395	0.079	0.669
*RMS AP*	<0.001	0.109	0.540	0.159	0.385
*RMS ML*	<0.001	0.084	0.637	0.070	0.702
*Ellipse Angle AP*	<0.001	−0.061	0.733	0.080	0.665
*Ellipse Angle ML*	<0.001	−0.134	0.452	−0.215	0.237
*95% Ellipse Area*	<0.001	0.049	0.782	0.092	0.617
*Ellipse Axis AP*	<0.001	0.110	0.536	0.143	0.435
*Ellipse Axis ML*	<0.001	−0.004	0.981	0.042	0.819
*Jerk AP*	<0.001	0.068	0.703	0.042	0.818
*Jerk ML*	<0.001	−0.125	0.481	−0.089	0.628
*SwayV AP*	<0.001	0.182	0.303	0.175	0.338
*SwayV ML*	<0.001	0.180	0.308	0.105	0.569
*SPathA AP*	<0.001	−0.006	0.973	−0.031	0.868
*SPathA ML*	<0.001	−0.209	0.236	−0.128	0.486

^1^ Features in italic were tested using the Spearman rank correlation, while the remainder were tested with Pearson correlation. Bolded values indicate significant correlation: * *p* < 0.05, ** *p* < 0.01.

**Table A3:** Correlation of BBS (Standing Feet Together) features with age (N = 49).

Feature ^1^	Normality	Correlation with Age		Controlled for Weight and Height	
	*p*	*r*	*p*	*r* *	*p* *
*F50% AP*	<0.001	−0.070	0.635	−0.133	0.374
F50% ML	0.101	−0.101	0.489	−0.097	0.517
*F95% AP*	0.001	−0.116	0.426	−0.188	0.207
F95% ML	0.162	**−0.312** *	0.029	**−0.305** *	0.037
*SC AP*	0.040	−0.067	0.647	−0.153	0.304
SC ML	0.121	−0.269	0.062	−0.261	0.076
*Max Acc AP*	<0.001	0.188	0.195	0.110	0.463
*Max Acc ML*	0.004	0.134	0.360	0.106	0.477
*Mean Acc AP*	0.003	0.101	0.492	0.163	0.273
*Mean Acc ML*	0.001	0.154	0.292	0.187	0.209
*RMS AP*	0.006	0.119	0.415	0.166	0.266
*RMS ML*	0.009	0.145	0.319	0.186	0.211
*Ellipse Angle AP*	<0.001	−0.023	0.876	−0.193	0.195
*Ellipse Angle ML*	<0.001	0.140	0.339	0.099	0.508
*95% Ellipse Area*	<0.001	0.164	0.259	0.149	0.318
*Ellipse Axis AP*	0.035	0.164	0.260	0.192	0.197
*Ellipse Axis ML*	<0.001	0.102	0.485	0.102	0.497
*Jerk AP*	0.025	−0.055	0.708	−0.108	0.469
Jerk ML	0.113	−0.170	0.244	−0.167	0.263
*SwayV AP*	<0.001	0.056	0.704	0.144	0.333
*SwayV ML*	<0.001	0.085	0.562	0.056	0.710
*SPathA AP*	0.029	−0.064	0.660	−0.110	0.461
SPathA ML	0.119	−0.169	0.244	−0.167	0.263

^1^ Features in italic were tested using the Spearman rank correlation; the remainder was tested with Pearson correlation. Bolded values indicate significant correlation: * *p* < 0.05, ** *p* < 0.01.

**Table A4:** Correlation of BBS (Standing Tandem) features with age (N = 49).

Feature ^1^	Normality	Correlation with Age		Controlled for Weight and Height	
	*p*	*r*	*p*	*r* *	*p* *
*F50% AP*	<0.001	−0.131	0.369	**−0.301** *	0.040
*F50% ML*	0.033	−0.045	0.760	−0.076	0.610
*F95% AP*	<0.001	−0.017	0.907	0.038	0.800
F95% ML	0.055	−0.050	0.733	−0.055	0.712
*SC AP*	0.032	−0.087	0.550	−0.152	0.308
SC ML	0.133	−0.076	0.605	−0.077	0.608
*Max Acc AP*	<0.001	0.096	0.510	−0.107	0.475
*Max Acc ML*	<0.001	0.031	0.834	−0.132	0.378
*Mean Acc AP*	<0.001	0.238	0.099	0.089	0.550
*Mean Acc ML*	<0.001	0.222	0.125	0.157	0.291
*RMS AP*	<0.001	0.163	0.264	0.024	0.870
*RMS ML*	<0.001	0.166	0.254	0.041	0.785
*Ellipse Angle AP*	<0.001	−0.070	0.634	−0.064	0.668
*Ellipse Angle ML*	0.040	0.070	0.631	0.049	0.746
*95% Ellipse Area*	<0.001	0.179	0.219	−0.042	0.779
*Ellipse Axis AP*	<0.001	0.113	0.441	−0.021	0.890
*Ellipse Axis ML*	<0.001	0.231	0.110	0.068	0.651
Jerk AP	0.137	0.172	0.238	0.189	0.203
Jerk ML	0.176	0.114	0.434	0.126	0.399
*SwayV AP*	<0.001	0.105	0.475	−0.016	0.917
*SwayV ML*	<0.001	−0.032	0.825	−0.071	0.635
SPathA AP	0.197	0.160	0.273	0.177	0.233
SPathA ML	0.323	0.106	0.471	0.118	0.430

^1^ Features in italic were tested using the Spearman rank correlation; the remainder was tested with Pearson correlation. Bolded values indicate significant correlation: * *p* < 0.05, ** *p* < 0.01.

**Table A5:** Correlation of BBS (Standing One Leg) features with age (N = 49).

Feature ^1^	Normality	Correlation with Age		Controlled for Weight and Height	
	*p*	*r*	*p*	*r* *	*p* *
*F50% AP*	<0.001	−0.015	0.98	0.084	0.577
F50% ML	0.106	−0.169	0.274	−0.155	0.299
F95% AP	0.100	0.014	0.922	0.029	0.848
*F95% ML*	<0.001	−0.103	0.479	−0.143	0.337
*SC AP*	0.042	0.037	0.800	0.052	0.726
SC ML	0.500	−0.175	0.228	−0.171	0.250
*Max Acc AP*	<0.001	0.185	0.202	0.139	0.353
*Max Acc ML*	<0.001	**0.432 ****	0.002	**0.348 ***	0.017
*Mean Acc AP*	<0.001	0.226	0.118	0.173	0.245
*Mean Acc ML*	<0.001	**0.361 ***	0.011	**0.377 ****	0.009
*RMS AP*	<0.001	0.226	0.118	0.166	0.264
*RMS ML*	<0.001	**0.356 ***	0.012	**0.379 ****	0.009
*Ellipse Angle AP*	<0.001	0.184	0.206	0.177	0.235
*Ellipse Angle ML*	0.018	−0.049	0.753	−0.029	0.848
*95% Ellipse Area*	<0.001	**0.292 ***	0.042	0.191	0.199
*Ellipse Axis AP*	<0.001	**0.354 ***	0.013	**0.316 ***	0.031
*Ellipse Axis ML*	<0.001	0.190	0.192	0.140	0.347
*Jerk AP*	<0.001	0.234	0.105	0.152	0.306
*Jerk ML*	<0.001	0.273	0.058	0.241	0.103
*SwayV AP*	<0.001	0.182	0.210	0.205	0.167
*SwayV ML*	<0.001	**0.283 ***	0.049	**0.324 ***	0.026
*SPathA AP*	<0.001	0.257	0.074	0.178	0.232
*SPathA ML*	<0.001	0.268	0.063	0.239	0.106

^1^ Features in italic were tested using the Spearman rank correlation; the remainder was tested with Pearson correlation. Bolded values indicate significant correlation: * *p* < 0.05, ** *p* < 0.01.

**Table A6:** Correlation of 10-Meter Walk Test (SSV) features with age (N = 49).

Feature ^1^	Normality	Correlation with Age		Controlled for Weight and Height	
	*p*	*r*	*p*	*r* *	*p* *
*Mean Vertical Displacement*	<0.001	−0.201	0.167	−0.177	0.234
Mean Stance Time	0.163	0.219	0.133	**0.313 ***	0.032
Mean Step Time	0.075	0.183	0.209	0.267	0.069
Mean Stride Time	0.073	0.179	0.219	0.541	0.264
*Mean Swing Time*	0.040	0.082	0.575	0.183	0.219
Mean Step Length	0.079	**−0.341 ***	0.017	**−0.367 ***	0.011
*Maximum Power Frequency*	<0.001	−0.221	0.127	−0.216	0.144
Stance Time Symmetry Ratio	0.826	−0.057	0.697	−0.049	0.742
*Step Length Symmetry Ratio*	<0.001	−0.152	0.296	−0.167	0.263
*Duration*	<0.001	0.235	0.104	**0.309 ***	0.034
Mean Velocity	0.116	**−0.658 ****	0.008	**−0.387 ****	0.007
N Steps	0.126	0.280	0.051	0.260	0.077
*Velocity Difference, FV – SSV*	<0.001	−0.013	0.931	−0.004	0.979

^1^ Features in italics were tested using the Spearman rank correlation; the remainder was tested with Pearson correlation. Bolded values indicate significant correlation: * *p* < 0.05, ** *p* < 0.01.

**Table A7:** Correlation of 10-Meter Walk Test (FV) features with age (N = 48).

Feature ^1^	Normality	Correlation with Age		Controlled for Weight and Height	
	*p*	*r*	*p*	*r* *	*p* *
Mean Vertical Displacement	0.383	−0.251	0.086	−0.272	0.067
Mean Stance Time	0.502	0.020	0.895	0.059	0.699
Mean Step Time	0.240	0.014	0.924	0.049	0.747
Mean Stride Time	0.248	0.012	0.936	0.048	0.753
Mean Swing Time	0.056	0.010	0.948	0.038	0.800
Mean Step Length	0.105	**−0.315 ***	0.029	**−0.370 ***	0.011
*Maximum Power Frequency*	0.003	**−0.355 ***	0.013	**−0.363 ***	0.013
Stance Time Symmetry Ratio	0.461	−0.078	0.598	−0.072	0.632
Step Length Symmetry Ratio	0.288	0.044	0.768	0.040	0.790
*Duration*	0.043	0.271	0.062	**0.327 ***	0.026
Mean Velocity	0.383	**−0.364 ***	0.011	**0.370 ***	0.011
N Steps	0.502	**0.365 ***	0.011	**0.367 ***	0.012

^1^ Features in italics were tested using the Spearman rank correlation; the remainder was tested with Pearson correlation. Bolded values indicate significant correlation: * *p* < 0.05, ** *p* < 0.01.

**Table A8:** Correlation of Timed Up and Go (TUG) features with age (N = 48).

Feature ^1^	Normality	Correlation with Age		Controlled for Weight and Height	
	*p*	*r*	*p*	*r* *	*p* *
SIT-TO-STAND					
Range Pitch Vel (i–ii)	0.408	**−0.307 ***	0.037	−0.290	0.051
Range Pitch Vel (ii–iii)	0.554	−0.212	0.148	−0.195	0.194
SD Pitch Vel (i–iii)	0.271	**−0.295 ***	0.042	−0.279	0.060
*Mean Pitch Vel (i–iii)*	0.045	**0.290 ***	0.046	**0.309 ***	0.036
Median Pitch Vel (i–iii)	0.222	0.132	0.372	0.110	0.466
Max Pitch Vel (i–ii)	0.825	**0.314 ***	0.030	**0.301 ***	0.042
Max Pitch Vel (ii–iii)	0.152	0.093	0.528	0.104	0.493
*Mean Pitch Acc (i–ii)*	<0.001	0.269	0.065	**0.391 ****	0.007
Mean Pitch Acc (ii–iii)	0.259	**−0.291 ***	0.045	−0.289	0.052
Mean Acc AP (i–iii)	0.182	−0.207	0.158	−0.203	0.176
SD Acc AP (i–iii)	0.192	−0.020	0.890	0.021	0.892
Median Acc AP (i–iii)	0.454	−0.085	0.563	−0.120	0.426
Duration (i–iii)	0.511	**0.352 ***	0.014	**0.344 ***	0.019
WALK					
RMS Acc AP	0.097	**0.378 ****	0.008	**0.366 ***	0.012
RMS Acc ML	0.145	0.110	0.455	0.103	0.495
*RMS Acc V*	0.047	**0.276 ***	0.058	**0.299 ***	0.043
Mean Step Time	0.491	0.022	0.884	0.034	0.822
*SD Step Time*	0.025	−0.120	0.418	−0.110	0.466
*N Steps*	0.003	**0.403 ****	0.005	**0.483 ****	0.001
*Duration*	<0.001	**0.432 ****	0.002	**0.488 ****	0.001
TURN 1					
Max Yaw Vel	0.397	−0.225	0.125	−0.213	0.154
*Mean Yaw Acc (i–ii)*	<0.001	−0.203	0.167	−0.181	0.228
*Mean Yaw Acc (ii–iii)*	<0.001	0.185	0.209	0.203	0.177
N Steps	0.460	−0.169	0.251	−0.187	0.212
Duration	0.217	0.100	0.500	0.094	0.535
TURN 2					
Max Yaw Vel	0.829	**−0.457 ****	0.001	**−0.455 ****	0.001
*Mean Yaw Acc (i–ii)*	<0.001	**−0.569 ****	<0.001	**−0.581 ****	<0.001
*Mean Yaw Acc (ii–iii)*	0.014	**0.366 ***	0.010	**0.315 ***	0.033
N Steps	0.947	**0.297 ***	0.040	**0.296 ***	0.046
Duration	0.246	**0.365 ***	0.011	**0.391 ****	0.007
STAND-TO-SIT					
*Range Pitch Vel (i–ii)*	0.026	−0.259	0.076	**−0.357 ***	0.015
Range Pitch Vel (ii–iii)	0.699	**−0.290 ***	0.045	**−0.303 ***	0.041
SD Pitch Vel (i–iii)	0.101	**−0.313 ***	0.030	**−0.335 ***	0.023
Mean Pitch Vel (i–iii)	0.362	−0.066	0.654	−0.084	0.581
Median Pitch Vel (i–iii)	0.761	−0.066	0.657	−0.097	0.521
*Max Pitch Vel (i–ii)*	0.002	0.213	0.146	**0.315 ***	0.033
Max Pitch Vel (ii–iii)	0.995	−0.223	0.127	−0.234	0.117
*Mean Acc Pitch (i–ii)*	0.001	**0.328 ***	0.023	**0.291 ***	0.050
*Mean Acc Pitch (ii–iii)*	0.002	**−0.362 ***	0.012	**−0.422 ****	0.004
Mean Acc AP (i–iii)	0.839	**−0.365 ***	0.011	**−0.398 ****	0.006
*SD Acc AP (i–iii)*	0.005	**−0.315 ***	0.029	**−0.326 ***	0.027
Median Acc AP (i–iii)	0.520	−0.259	0.076	−0.279	0.060
*Duration (i–iii)*	<0.001	0.226	0.122	0.238	0.112

^1^ Features in italics were tested using the Spearman rank correlation; the remainder was tested with Pearson correlation. Bolded values indicate significant correlation: * *p* < 0.05, ** *p* < 0.01.
